# Leksell Gamma Knife : An Effective Non Invasive Treatment for Rare Case of Nelson’s Syndrome

**DOI:** 10.5812/ijem.10225

**Published:** 2013-07-01

**Authors:** Faheem Arshad, bashir ahmad Laway, Manzoor Ahmad bhat, hakim Irfan Showkat, suman Kotwal, shahnaz Ahmad Mir

**Affiliations:** 1 Department of Endocrinology, Sher-i-Kashmir Institute of Medical Sciences Soura, Resident Scholar Endocrinology, Srinagar, India

**Keywords:** Nelson’s Syndrome, Cushing’s Disease, Adrenalectomy

## Abstract

Nelson’s syndrome nowadays a rare entity results from an adrenocorticotropin (ACTH)–secreting pituitary adenoma in patients with refractory Cushing's disease after a therapeutic bilateral adrenal gland removal. We report a case of 25 year old female with cushing’s disease who was initially managed with medical treatment, but in view of severe persistent hyper cortisol state was subjected to bilateral adrenalectomy following which she developed Nelson’s syndrome after a gap of six years, which was difficult to diagnose because of limited investigations available. Patient was managed with stereotactic radiosurgery (gamma knife surgery).

## 1. Introduction

Nelson’s syndrome, an uncommon life-threatening complication after complete removal of both adrenals for the treatment of Cushing’s disease, is one of the most difficult to manage of all endocrine conditions. Despite a declining trend in mortality rates, this syndrome has been seen to have significant morbidity (1). This syndrome is rare nowadays due to less use of total adrenalectomy as a procedure for treatment of refractory cushing’s disease . Patients with this syndrome usually present with clinical features of an enlarging pituitary tumor which include defects in visual field and cranial nerve involvement, compressive effects and invasion into surrounding structures due to tumor itself. Although these manifestations are less common for presentation in modern times, but increased pigmentation of skin and mucous membranes (particularly on extensor surfaces, flexures, over scars and on the areolae) remains an important clinical finding. Other presentations include headaches, diabetes insipidus, panhypopituitarism, pituitary apoplexy, testicular pain and oligospermia.

We report a case of Nelson's syndrome who presented with enlarging pituitary growth involving the cavernous sinus associated with elevated fasting plasma ACTH levels and hyperpigmentation with history of total adrenalectomy for refractory cushing’s disease.

## 2. Case Report

25 year female presented to our tertiary care hospital with complaints of sudden onset severe global headache, drooping of right eye lid and diplopia and vomiting few episodes. She denied any history of fever, dysentery, abdominal pain, trauma, abnormal movements, loss of consciousness or fever. On enquiring, the patient gave history of longstanding ill health in 2004, with one year of progressive weight gain, amenorrhea, acne, hair growth and difficulty getting up from sitting position with her investigations revealing high evening cortisol , loss of diurnal variation abnormal low dose dexamethasone suppression test, high basal ACTH ( ACTH dependent) and more than 90 % suppression of cortisol after high-dose dexamethasone suppression test (Table 1), ectopic cushing’s was ruled out with MRI revealing microadenoma [0.8×0.7×0.8 cm] no suprasellar or parasellar extension.

**Table 1.** Hormonal Profile

Hormone	Value	Normal Value
**8 am cortisol**	30.34 µg/dL	5-25
**11 pm cortisol**	19.57 µg/dL	0-10
**LDST**	17.23 µg/dL	< 1.8
**HDST**	3.56 µg/dL	-
**Serum ACTH**	24 pg/ mL	10-46
**Prolactin**	14.33 ng/mL	< 28
**T 4**	7.43 µg/dL	5.5 - 10
**TSH**	4.65 µIU/ mL	0-5

Abbreviations: HDST, high dose dexamethasone suppression test; LDST, low dose dexamethasone suppression test

CT scan adrenals was normal, and a diagnosis of cushing’s disease was made in 2004. Subsequently, she was managed with medical treatment in form of Ketoconazole and then subjected to transsphenoidal surgery with histopathology revealing normal pituitary. There was transient improvement in her symptoms but she had a persistent hypercortisol state presenting in form of difficulty in walking, low back ache, continuation of amenorrhea, weight gain and worsening of proximal muscle weakness and investigations revealing a derranged hormonal profile and MRI hip showing bilateral avascular necrosis of the femur neck (for which she was managed by bilateral core decompression). MRI pituitary before adrenalectomy was done, which revealed partial empty sella. She was subjected to total bilateral adrenalectomy (TBA) in december 2005. HPE of adrenals revealed normal adrenal tissue. Immediate post TBA ACTH was 46 Pg/ mL. This was followed by marked improvement in her symptoms until 2011 when she presented with above mentioned symptoms.

**Figure 1.**
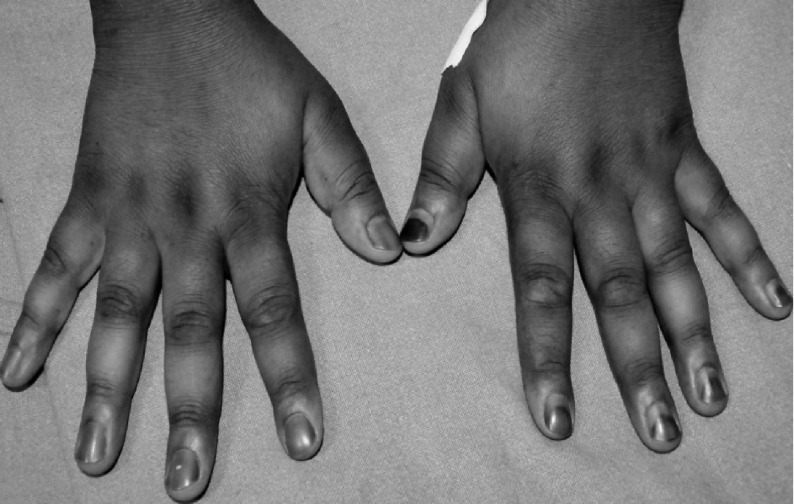
Showing Diffuse Hyperpigmentation Involving Extensor Surfaces of Hands

Her physical examination revealed an ill-looking, pulse of 74 beats/ minute, blood pressure of 130/80 mm Hg without any postural drop, respiratory rate of 18/ minute, and oral temperature of 97.6°F (36.4°C). The patient had mild pallor with severe diffuse pigmentation involving face, neck, bucal mucosa, extensor surface of hands and feet (Figure 1).

**Figure 2.**
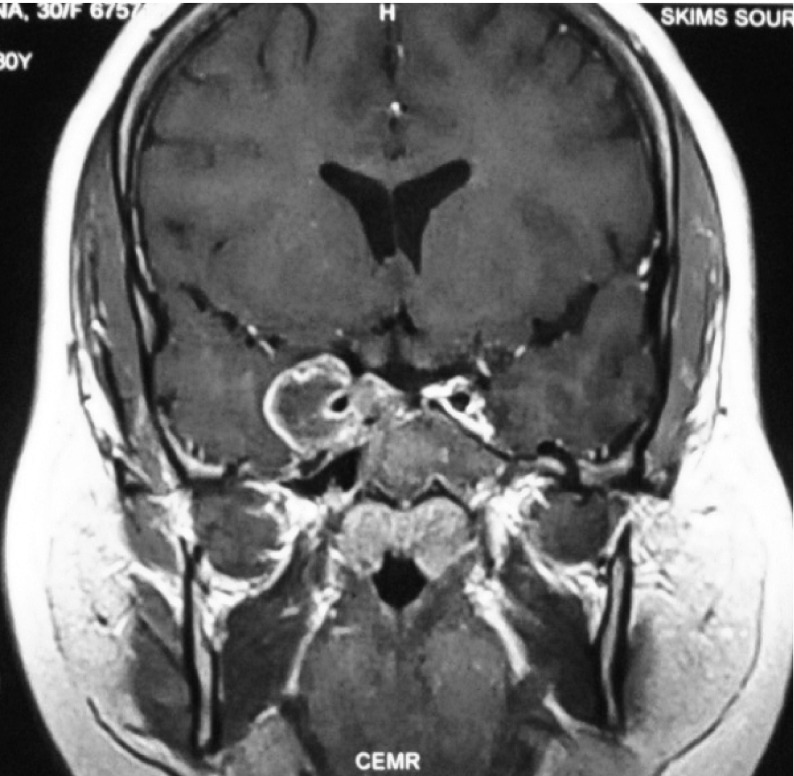
T1 Weighted (Gadolinium enh.) MRI Revealing Large Irregular Sellar Mass With Parasellar Extension With Infiltration of Cavernous Sinus and Encasement of Internal Carotid Artery.

Respiratory system, Cardiovascular system and abdominal examinations were non-contributory. Central nervous system examination revealed the right side complete ptosis with reduced ocular movement nasally; left eye reduced ocular motility temporally suggesting homonymous hemianopia. Laboratory evaluation revealed a haemoglobin concentration was 11.3 g/dL and the white cell count 7.3×109/L, with 73% neutrophils, and 22% lymphocytes. Kidney function test revealed urea 38 mg/dL and creatinine 0.68 mg/dL. Liver function test was normal. Lipid profile was deranged revealing total cholesterol 262 mg/dL, triglycerides 193 mg/dL, low density lipoprotein levels (LDL) 190.4 mg/dL and high density lipoprotein levels (HDL) 33mg/dL. Her hormonal profile revealed prolactin < 1 ng/mL (normal range 1-27) , thyroxine 4.05 µg/dL (4-13), thyroid-stimulating hormone (TSH) < 0.15 µIU/mL (0.5-6.5) and adrenocorticotropin hormone (ACTH) levels 206 pg/ mL (10-46). Her 8 am cortisol levels were 6.2 µg/dL (5-25). Due to financial constraints MRI was delayed but later after arranging finances patient was subjected to magnetic resonance imaging (MRI) brain with sellar and parasellar focus which revealed large irregular sellar mass with parasellar extension (especially right side) with infiltration of cavernous sinus and encasement of internal carotid artery (Figure 2). The diagnosis of Nelson’s syndrome with external ophthalmoplegia with right sided ptosis was made on THE basis of her history, present complaints, high ACTH levels and MRI brain.

**Figure 3.**
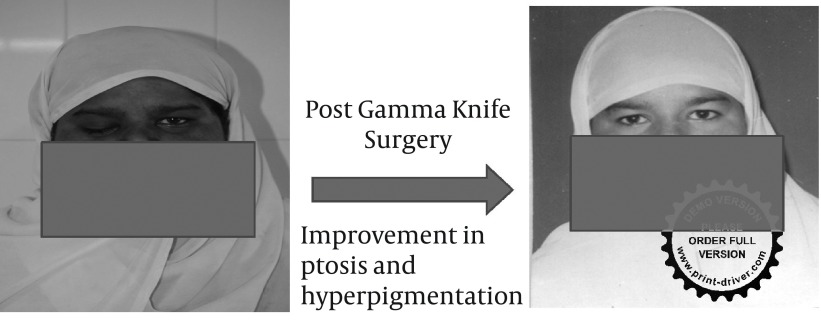
Comparison Between Pre and Post Gamma Knife Surgery Images of Patient.

The patient was managed initially with hydrocortisone, thyroxine, parenteral analgesics and anti-emetics. Subsequently, she was subjected to gamma knife surgery. Post gamma knife surgery she marked improvement in her symptoms especially diffused pigmentation and ptosis which can be appreciated from pre and post gamma knife surgery photographs of the patient (Figure 3).

## 3. Discussion

The present report describes a rare case of Nelson’s syndrome, which results from an adrenocorticotropin (ACTH) secreting pituitary adenoma in patients with Cushing's disease after a therapeutic bilateral adrenalectomy (TBA). It is a complication of treatment of intractable Cushing’s disease with reported incidence of 8–43% in adults and 25–66% in children (1). Nelson’s syndrome has been seen to occur as late as 24 years after total adrenalectomy (range two months–24 years), therefore all patients of intractable cushing’s disease may need life long pituitary imaging following TBA treatment of difficult Cushing’s disease, and any increase in tumour size is significant (2). It was first time described in 1958 by Don Nelson *et al*. who reported the first case of Nelson’s syndrome in a 33-year-old woman, she developed this syndrome 3 years after being operated for intractable cushing’s disease. Her presentations were visual field defects, skin hyperpigmentation, raised plasma ACTH and a large sellar mass shown on skull X-ray which was a pituitary tumor, and its removal led to resolution of symptoms (3). Our patient developed features of Nelson’s syndrome after a gap of seven years. Although definitive diagnostic criteria for Nelson’s syndrome are lacking, Barbar, et al. proposed a new criteria which included an elevated 0800 h plasma level of ACTH (> 500 ng/L) our patient had high ACTH but less as per Barbar’s criteria because of the fact that ACTH being a volatile molecule disintegrates rapidly, so adequate precautions need to be taken when samples of ACTH are taken (1). The most reliable biochemical feature of Nelson’s syndrome is a marked rise of plasma ACTH, which continues to rise after adrenalectomy, as was seen with our patient (4). Radiological diagnosis of Nelson’s syndrome included enlarging pituitary mass of which MRI is the best imaging modality, detecting tumours as small as 3 mm (5). We had difficulty in diagnosing this case as previous images were not available so as to compare them with the new images to diagnose nelson's syndrome syndrome as per Barber’s criteria. So with clinical knowledge and limited investigations available, a diagnosis of Nelson’s syndrome was made. Athough a number of factors have been reported which would predict the onset of development Nelson’s syndrome and influence further progression but constancy of these factors have not been established. The etiology of Nelson’s syndrome is also partially understood. One hypothesis is that a loss of the suppressive negative-feedback effects of elevated endogenous cortisol levels on corticotroph cells following TBA, with concurrent recovery of hypothalamic CRH production is sufficient to result in progressive corticotroph neoplasia in some patients (6). Treatment of Nelson’s syndrome is very difficult. First-line treatment option is pituitary surgery particularly if there is involvement of the optic apparatus with success rates ranging from 10 to 70% (7). Despite surgical intervention in some patients, progression of Nelson’s syndrome occured and adjuvant radiotherapy may be needed in up to 30% cases. The role of administering adjuvant radiotherapy to be pituitary for all Cushing's disease patients following TBA surgery is limited (8) but neo-adjuvant radiotherapy has been reported to decrease the incidence of Nelson’s syndrome. Limited data on the use of gamma knife surgery (GKS) in Nelson's syndrome are conflicting. In one study of Nelson's syndrome patients showed no tumor re-growth at seven years post GKS therapy, another study reported remission rates post GKS to be only 14% (9). Our patient did well with gamma knife surgery and had marked improvement in symptoms esp. diffuse pigmentation and ptosis. Medical management in form of selective somatostatin analogues, peroxisome proliferator-activated receptor γ agonists, dopamine agonists, sodium valproate, and temozolomide have been tried, but of limited benefit .One case report of an aggressive Nelson’s tumour, which failed to respond to both surgical and radiotherapy (including gamma knife treatment), showed an excellent response to temozolomide with a significant reduction in plasma ACTH (10).

Our case was a refractory cushing’s disease, after treatment leading to Nelson’s syndrome which is rare and was difficult to diagnose because of limited investigations available and difficult to treat because of limited management options. But our patient was managed effectively by gamma knife surgery.

## 4. Conclusions

Nelson’s syndrome, a rare case, can be diagnosed with a proper assessment and clinical judgment even with limited investigations available. Only laboratory parameter which could guide for diagnosis is ACTH level, which should be measured in all patients following bilateral removal of adrenal glands. Gamma knife surgery proved to be very effective therapy in our patient as there was marked improvement in her symptoms.
